# Transmission of Curing Light through Moist, Air-Dried, and EDTA Treated Dentine and Enamel

**DOI:** 10.1155/2016/5713962

**Published:** 2016-06-30

**Authors:** E. Uusitalo, J. Varrela, L. Lassila, P. K. Vallittu

**Affiliations:** ^1^Institute of Dentistry, University of Turku, 20520 Turku, Finland; ^2^City of Turku Division of Welfare, 20520 Turku, Finland; ^3^Department of Oral Development and Orthodontics, University of Turku, 20520 Turku, Finland; ^4^Department of Biomaterials Science and Turku Clinical Biomaterials Centre (TCBC), 20520 Turku, Finland

## Abstract

*Objective*. This study measured light transmission through enamel and dentin and the effect of exposed dentinal tubules to light propagation.* Methods*. Light attenuation through enamel and dentin layers of various thicknesses (1 mm, 2 mm, 3 mm, and 4 mm) was measured using specimens that were (1) moist and (2) air-dried (*n* = 5). Measurements were repeated after the specimens were treated with EDTA. Specimens were transilluminated with a light curing unit (maximum power output 1869 mW/cm^2^), and the mean irradiance power of transmitting light was measured. The transmission of light through teeth was studied using 10 extracted intact human incisors and premolars.* Results*. Transmitted light irradiance through 1 mm thick moist discs was 500 mW/cm^2^ for enamel and 398 mW/cm^2^ for dentin (*p* < 0.05). The increase of the specimen thickness decreased light transmission in all groups (*p* < 0.005), and moist specimens attenuated light less than air-dried specimens in all thicknesses (*p* < 0.05). EDTA treatment increased light transmission from 398 mW/cm^2^ to 439 mW/cm^2^ (1 mm dentin specimen thickness) (*p* < 0.05). Light transmission through intact premolar was 6.2 mW/cm^2^ (average thickness 8.2 mm) and through incisor was 37.6 mW/cm^2^ (average thickness 5.6 mm).* Conclusion*. Light transmission through enamel is greater than that through dentin, probably reflecting differences in refractive indices and extinction coefficients. Light transmission through enamel, dentin, and extracted teeth seemed to follow Beer-Lambert's law.

## 1. Introduction

Light transmission through human tooth became a matter of interest when resin based light cured orthodontic adhesives spread in common use and due to the increased use of indirectly luted restorations which are bonded with dual-curing resin composite luting cements [1]. Indirect luting is used especially in prosthodontics but could also be used in orthodontics. In dual-curing resin, composite light is typically used to coinitiate polymerization and in order to achieve proper curing, light irradiation through the curing process is often needed. Polymerization occurs when free radicals are generated, and this can be due to chemical process or curing light initiation [2]. Ceramic inlays, onlays, and veneers are usually bonded with dual-curing cements, which requires light transmission through the ceramic material. In dual-curing resins the cement includes chemical activator, which increases the amount of free radicals when the curing light is insufficient and photoinitiators are unable to produce free radicals required for polymerization [3]. Because the light initiated adhesives are also used in orthodontics to bond brackets, it is important to study the light transmission through the tooth to evaluate the possibility of light curing with transillumination.

Light curing adhesives are used under orthodontic appliances, such as stainless steel brackets that do not let any light pass through [4]. A common way to cure the adhesive under the bracket is to cure 20 s from both mesial and distal side of the bracket. This method may provide clinically satisfactory bond strengths, but there is a risk that the adhesive is polymerized only from the edges of the bracket leaving the center incompletely polymerized [5]. Furthermore the curing procedure of the adhesive under the bracket, especially in the posterior area, might be difficult to execute.

To solve this problem it was suggested by Tavas and Watts [6] that light curing resin based adhesive could be cured with transillumination through the tooth [6, 7]. To achieve a satisfactory degree of monomer conversion and bond strength, it has been shown that the curing time must be prolonged [8, 9] because of the dentin and enamel barrier. Oesterle and Shellhart [9] presented in their study that a 10 s increase in transillumination time, total curing time being 50 s, enhances the bracket bond strength comparable to labial curing. However extended curing times have a risk of temperature rise in the pulp chamber [10], and the risk is also proportional to the power output of the light curing unit [10, 11].

Light transmission through dentin and enamel is not well known. It has not been reported in the literature whether the hard tissues of the tooth follow the Beer-Lambert law. The Beer-Lambert law is the relation between absorbance and material concentration, but the linearity is limited if the media is highly scattering, as it is in the case of enamel and dentin. It has been suggested that light scatters mainly from the dentin tubules when light irradiation is applied parallel to the dentin tubules [12]. On the other hand, it has been shown that the obliterated dentin tubules do not significantly affect light transmission through dentin [13, 14]. Most studies are only concerned with the optical properties of dentin, but in the orthodontic field teeth are usually intact so the enamel's optical properties must also be taken into consideration. Light transmission through enamel is less well known, and the existing studies consider the light scattering effect only from the esthetic standpoint.

The aim of this study was to measure the light irradiance attenuation of curing light in respect to test specimen thickness for both enamel and dentin.

## 2. Material and Methods

Teeth used in this study were extracted human third molars, premolars, and incisors, restored in a Chloramine-T/distilled water solution. Teeth were sound without visually detected cracks, caries, or dental fillings. Forty third molars were vertically cut in the buccolingual direction into slices with a thickness of 1 mm to obtain 20 enamel specimens and 20 dentin specimens. The test specimens were cut so that the enamel specimens contained only enamel and the dentin specimens only dentin without visible remnants of any other tissue, and the average diameter of the round shaped test specimens was 5,5 mm (SD 0,58). The specimens were cut with histological saw (Secotom-50, Struers A/S, Ballerup, Denmark) and finished on a polishing machine (LaboPol-1, Struers A/S, Ballerup, Denmark) using 500 grit-SiC paper. After preparing the test specimens they were stored in oil-free distilled water.

The experiment was carried out by measuring light attenuation through 1 mm, 2 mm, 3 mm, and 4 mm of enamel and through 1 mm, 2 mm, 3 mm, and 4 mm of dentin (*n* = 5 in each). Transmitted irradiance was measured first when the specimens were moist, and the measurements were repeated when the specimens were gently air-dried. The increase in thickness was executed by piling the specimens on the sensor. Piled test specimens were picked up randomly among 20 existing specimens. The tip of the light curing unit (LCU) was held as close as possible to the specimen on the sensor. The maximum power output of the light curing unit (led emitting diode, Elipar*™* S10, 3 M ESPE, St. Paul, MN, USA) used in this study was 1869 mW/cm^2^ when set fully against the sensor, with a center wavelength of 455 nm ± 10 according to the manufacturer. The mean irradiance of transmitted light was measured by a MARC**®** spectrometer and analyzed using BlueLight® (MARC Resin Calibrator, BlueLight analytics inc., Halifax, Nova Scotia, Canada).

After measuring light transmittance, the enamel and dentin specimens were treated with 19.5% EDTA ethylenediaminetetraacetic acid (File Eze® 19%, Ultradent Products, Inc., 505 West 10200 South Jordan, UT 84095) for 1 minute on both sides to remove the smear layer in order to expose the dentin tubules and enamel rods. After EDTA treatment, light transmittance was measured as described previously, through 1 mm thick enamel and dentin specimens, first as moist and then as air-dried.

Ten extracted human incisors and ten premolars (extracted mainly for orthodontic reasons) were used to study light attenuation through teeth. Each tooth was placed with its labial surface facing the sensor (Ø 4 mm) and the LCU tip on the lingual surface. The thickness of each tooth was measured at the thickest point of the crown, perpendicular to the longitudinal axis of the tooth with a digital slide gauge (Vernier, Millikan Way, Beaverton), with an accuracy of 0.02 mm. The standardized distance of the LCU tip from the sensor was 10 mm for the premolars and 7 mm for the incisors.

Data was analyzed with SPSS (IBM SPSS Statistics for Windows, Version 21.0. Armonk, NY), using a preset level of statistical significance of *p* < 0.05. The normally distributed data was analyzed using Pearson correlation coefficient, a two-way analysis of variance (ANOVA), and Tukey's* post hoc* test. In the ANOVA, the transmitted irradiance (mW/cm^2^) was the dependent variable and the factors were thickness and moist/air-dried specimen and their interactions.

## 3. Results

**Figure 1:**
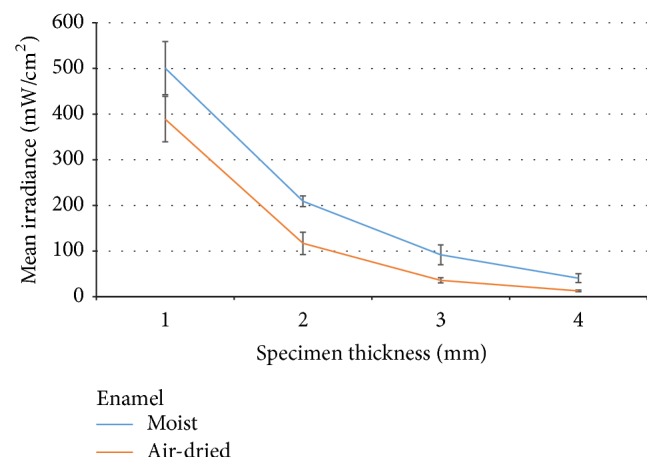
Mean irradiance (mW/cm^2^) through enamel specimens at different thicknesses.

**Figure 2:**
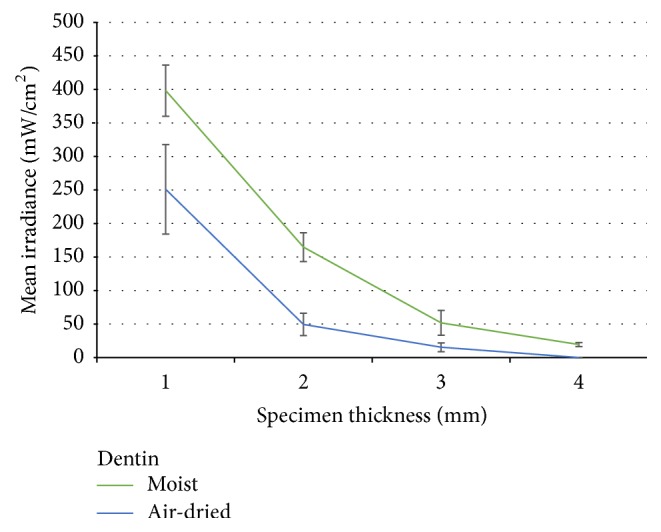
Mean irradiance (mW/cm^2^) through dentin specimens at different thicknesses.

The two-way ANOVA revealed a significant difference in light attenuation between enamel and dentin (*p* < 0.005) and also in moist/air-dried specimens (*p* < 0.05) as shown in Figures 1 and 2. The increase in test specimen thickness significantly affected the mean irradiance (mW/cm^2^) of transmitting light (*p* < 0.005), and for the 4 mm dentin group, the transmitted irradiance was below the level of detection. The values of mean irradiances are presented in Table 1 among Tukey's* post hoc* test results.

**Figure 3:**
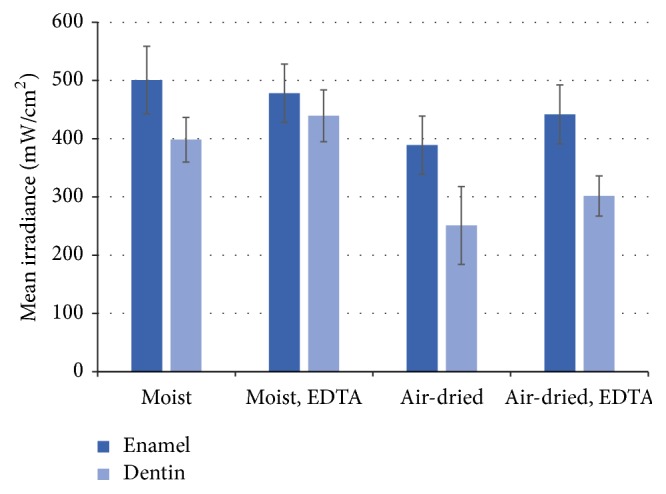
Mean irradiance (mW/cm^2^) through 1 mm specimens before and after EDTA treatment. Vertical bars demonstrate standard deviation.

After EDTA treatment, the transmission of curing light through 1 mm thick test specimens was significantly higher (*p* < 0.05), except in the moist enamel group where the difference was not statistically significant (Figure 3). A two-way ANOVA revealed statistically significant differences between enamel and dentin (*p* < 0.05), moist or air-dried (*p* < 0.05), and whether the test specimens were treated with EDTA or not (*p* < 0.05). However due to a wide standard deviation, there was no statistical difference in the increase of transmitted light intensity between enamel and dentin when treated with EDTA.

**Figure 4:**
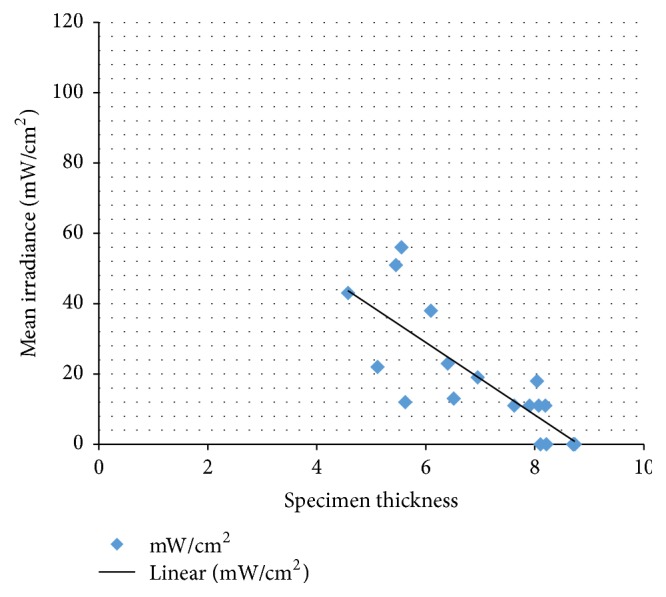
Mean irradiances (mW/cm^2^) through incisors and premolars.

Thicknesses and mean irradiances (mW/cm^2^) of light through incisors and premolars are presented in Table 2. Light transmitted somehow through incisors, but transmission through premolars was not significant, with the highest irradiance of 18 mW/cm^2^. Incisors and premolars were measured moist. The regression analysis was done to calculate the correlation between incisors and premolars. The coefficient of determination was *R* ^2^ = 0.65 and the correlation coefficient was *r* = 0.81 (*p* < 0.001) (Figure 4).

## 4. Discussion

In this study it was demonstrated that light transmission is considerably influenced when there is dentin or enamel between the light source and a light irradiance power detector. This issue has relevance when light curing adhesives and resin composites are cured through dentin and enamel, which can be the case in orthodontic applications. It has been shown that some dual-curing resin composite luting cements require light irradiance to reach an adequate degree of monomer conversion [1]. Thus, the findings of this study have relevance also for restorative dentistry and adhesive prosthodontics. The direction of light transmission in relation to microstructure of dentin and enamel varies in different applications.

Vaarkamp et al. [12] suggested that the tubules are the main cause of light scattering in dentin, because the light transmission was more intense when the dentin was irradiated parallel to dentin tubules. However, earlier studies concerning light transmission through dentin were performed with laser light that has been largely replaced by curing using LED light. Propagation of curing light through a highly scattering media may differ when different light sources are being used. In this study all the specimens were cut in the same direction, so there is no variation between specimen size and shape that could cause alteration to the results. All the dentin discs were cut near the dento-enamel junction; thus the variation in tubule size, shape, and amount may vary because of the physical alteration.

Enamel reflects the wavelength of blue light in certain directions [15], which is due to anisotropy of the enamel [16]. The wavelength of blue light is relatively low (450–495 nm), and it has been observed that translucency increases as the wavelength of the light increases [17]. Light propagates through enamel also by scattering along enamel rods and hydroxyapatite crystals, and the refractive index for enamel is 1.63 and 1.54 for dentin [18]. When enamel specimens were air-dried, the light transmittance decreased significantly, because the water around the enamel rods was replaced with air. The refractive index for water is 1.33 and for air is 1.00, which explains the difference in light attenuation. The different refractive indexes of water and air may have influenced the results, because of the scattering from the water between the piled test specimens. The findings from this study support the results of Brodbelt et al. [19], because the light transmission reverted when the specimens were rewetted.

When light propagates through turbid media (scattering media), the light is composed of absorbed, transmitted, and reflected light [20]. However, the absorbance values are hard to approximate from the Beer-Lambert law, because scattering cannot be separated and considered as an independent phenomenon [21]. Based on the results of this study, it can be suggested that the light attenuation through enamel and dentin follows Beer-Lambert law within the used wavelength of blue light that was used in this study. In case of enamel, reflected light is the wavelength of blue light and is called Rayleigh scattering. Rayleigh scattering appears when light scatters from electrically polarized particles that are minor to the wavelength of light resulting in the scattering which is visible blue light [22]. The scattering of curing light is also affected by the specimen surface texture. The test specimens were polished with the 500 grit-SiC paper, and some of the test specimens were treated with 19.5% EDTA. The EDTA treated surfaces are presumably rougher than the ones polished with 500 grit-SiC paper, so the scattering from the test specimen surface may have been influenced. Edge-loss effect may have influenced the results, since there was no mold to inhibit scattering to the edges [23]. This should be taken into consideration when doing further research about optical properties of a tooth.

In this study the smear layer was removed with EDTA from 1 mm thick test specimens after present studies had been executed, and the light transmittance was then measured again. The reason why the EDTA treatment was not performed on thicker specimens was due to the inability to create a continuous tubule structure with a piling technique. The EDTA treatment did not significantly affect light propagation through dry enamel, which was predictable; hence the hydroxyapatite crystals contribute light propagation through enamel the most, rather than the enamel rods [12]. Hence the light attenuation was only 2% greater when the dentin tubules were obliterated, the results of this study support the findings of Turrioni et al. [13] and Kienle et al. [14], who suggest that the light propagation through dentin is a result of scattering of intertubular dentin. The specimens in this study are cut from the different axis of the tooth than ones in previously mentioned studies, so that may explain the difference between results in light attenuation. To achieve greater clinical relevance, the influence of the resin adhesive system to the light attenuation through tooth needs further studies. This is relevant especially when the light curing is executed from the palatal/lingual side of the tooth, so that the curing light has to penetrate also through adhesive resin system.

The difficulty in metal bracket bonding is obtaining a satisfactory degree of conversion when using light initiated resin based adhesives. However, it has been observed that the light curing adhesives provide better bond strengths than adding a chemical initiator to the adhesive [23]. It has been also revealed that when using dual-curing adhesives, the degree of conversion is significantly higher when the adhesive is light-cured than auto-cured [24]. These studies are performed with ceramic composites, so that the light curing is performed through 3 mm thick composite blocks. However, the optical properties of translucent composites differ from the optical properties of human tooth [25]. The results of this study offer systematic information about light attenuation through human tooth and can therefore be used when evaluating alternative curing modes for orthodontic adhesives.

Since the output power of light curing units has increased, the curing times have diminished. The energy required to cure the resin is approximately 16 J/cm^2^ [26], so with an output power of 1900 mW/cm^2^ the required curing time is 8.4 s. According to the measurements of extracted human incisors and premolars, bonding brackets by transillumination would require a 426 s curing time to reach the energy of 16 J/cm^2^. It can be assumed that light propagation through vital teeth is greater, due to the scattering effect of blood and nervous tissue. Even though light attenuates greatly when transmitting through the entire tooth, there are studies that show clinically satisfactory bond strengths when orthodontic brackets are bonded using the transillumination technique [7–9]. This may be due to light scattering from the edges of the tooth, where the enamel/dentin barrier is minor compared to that through the thickest point of the tooth. In this study the sensor was placed so that the light transmission was measured only from the thickest point of the tooth. Further investigation is needed to study the bond strengths and the degree of conversion when light curing of the resin based adhesive is performed with transillumination through tooth and when cured indirectly from the mesial and distal side of the bracket.

## 5. Conclusions

- Light transmission through enamel and dentin seems to follow Beer-Lambert's law in the wavelength of blue light.
- Light transmission through dentin was less than that through enamel.
- Moist dentine and enamel transmitted light better than air-dried counterparts.
- The exposed dentin tubules enhanced the light transmission through dentin.

## Figures and Tables

**Table 1:** The average mean irradiance and standard deviations (mW/cm^2^) of each group, where *n* = 5. Superscripts describe statistical difference between groups.

Specimen thickness	Enamel		Dentin	
	Moist	Air-dried	Moist	Air-dried
1 mm	500,6 (58,0)^A,a^	389 (49,9)^B,a^	398,2 (38,4)^C,a^	251 (66,7)^D,a^
2 mm	209,2 (11,6)^A,b^	117 (24,4)^B,b^	164,8 (21,6)^C,b^	49,4 (16,8)^D,b^
3 mm	92 (21,8)^A,c^	36 (5,5)^B,c^	51,8 (18,5)^C,c^	15,4 (6,6)^D,c^
4 mm	40,8 (9,7)^A,d^	12,8 (1,9)^B,d^	19,4 (3,1)^C,d^	0 (0)^D,d^

**Table 2:** Average thicknesses and mean irradiances of incisors and premolars.

	Incisors	Premolars
Specimen thickness	5,6 mm (SD 0,91)	8,2 mm (SD 0,37)
Mean irradiance (mW/cm^2^)	37,6 (SD 26,6)	6,2 (SD 6,9)
